# Probing the CRL4^DCAF12^ interactions with MAGEA3 and CCT5 di-Glu C-terminal degrons

**DOI:** 10.1093/pnasnexus/pgae153

**Published:** 2024-04-10

**Authors:** Germanna Lima Righetto, Yanting Yin, David M Duda, Victoria Vu, Magdalena M Szewczyk, Hong Zeng, Yanjun Li, Peter Loppnau, Tony Mei, Yen-Yen Li, Alma Seitova, Aaron N Patrick, Jean-Francois Brazeau, Charu Chaudhry, Dalia Barsyte-Lovejoy, Vijayaratnam Santhakumar, Levon Halabelian

**Affiliations:** Structural Genomics Consortium, University of Toronto, Toronto, Ontario M5G 1L7, Canada; Department of Pharmacology and Toxicology, University of Toronto, Toronto, Ontario M5S 1A8, Canada; Structural and Protein Sciences, Therapeutics Discovery, Janssen Research and Development, Spring House, PA 19044, USA; Structural and Protein Sciences, Therapeutics Discovery, Janssen Research and Development, Spring House, PA 19044, USA; Structural Genomics Consortium, University of Toronto, Toronto, Ontario M5G 1L7, Canada; Structural Genomics Consortium, University of Toronto, Toronto, Ontario M5G 1L7, Canada; Structural Genomics Consortium, University of Toronto, Toronto, Ontario M5G 1L7, Canada; Structural Genomics Consortium, University of Toronto, Toronto, Ontario M5G 1L7, Canada; Structural Genomics Consortium, University of Toronto, Toronto, Ontario M5G 1L7, Canada; Structural Genomics Consortium, University of Toronto, Toronto, Ontario M5G 1L7, Canada; Structural Genomics Consortium, University of Toronto, Toronto, Ontario M5G 1L7, Canada; Structural Genomics Consortium, University of Toronto, Toronto, Ontario M5G 1L7, Canada; Discovery Technology and Molecular Pharmacology, Therapeutics Discovery, Janssen Research and Development, LLC, Welsh and McKean Roads, Spring House, PA 19477, USA; Discovery Chemistry, Therapeutics Discovery, Janssen Research and Development, LLC, 3210 Merryfield Row, La Jolla, CA 92121, USA; Discovery Technology and Molecular Pharmacology, Therapeutics Discovery, Janssen Research and Development, LLC, Welsh and McKean Roads, Spring House, PA 19477, USA; Structural Genomics Consortium, University of Toronto, Toronto, Ontario M5G 1L7, Canada; Department of Pharmacology and Toxicology, University of Toronto, Toronto, Ontario M5S 1A8, Canada; Structural Genomics Consortium, University of Toronto, Toronto, Ontario M5G 1L7, Canada; Structural Genomics Consortium, University of Toronto, Toronto, Ontario M5G 1L7, Canada; Department of Pharmacology and Toxicology, University of Toronto, Toronto, Ontario M5S 1A8, Canada

**Keywords:** E3 ligase, PROTAC, DCAF12, MAGEA3, CCT5

## Abstract

Damaged DNA-binding protein-1 (DDB1)- and CUL4-associated factor 12 (DCAF12) serves as the substrate recognition component within the Cullin4–RING E3 ligase (CRL4) complex, capable of identifying C-terminal double-glutamic acid degrons to promote the degradation of specific substrates through the ubiquitin proteasome system. Melanoma-associated antigen 3 (MAGEA3) and T-complex protein 1 subunit epsilon (CCT5) proteins have been identified as cellular targets of DCAF12. To further characterize the interactions between DCAF12 and both MAGEA3 and CCT5, we developed a suite of biophysical and proximity-based cellular NanoBRET assays showing that the C-terminal degron peptides of both MAGEA3 and CCT5 form nanomolar affinity interactions with DCAF12 in vitro and in cells. Furthermore, we report here the 3.17 Å cryo-EM structure of DDB1–DCAF12–MAGEA3 complex revealing the key DCAF12 residues responsible for C-terminal degron recognition and binding. Our study provides new insights and tools to enable the discovery of small molecule handles targeting the WD40-repeat domain of DCAF12 for future proteolysis targeting chimera design and development.

**Significance Statement**

The ubiquitin–proteasome system is a major pathway for degrading and eliminating misfolded or regulatory proteins in eukaryotic cells. Damaged DNA-binding protein-1 (DDB1)- and CUL4-associated factor 12 (DCAF12) is the substrate recognition component of the Cullin4–RING E3 ligase that was recently shown to recognize C-terminal double-glutamic acid residues of melanoma-associated antigen 3 (MAGEA3) and T-complex protein 1 subunit epsilon (CCT5) to promote degradation. In this study, we characterized the MAGEA3 and CCT5 C-terminal degron interactions with DCAF12 biophysically and in proximity-based cellular NanoBRET assays. We also generated the Cryo-EM structure of DDB1–DCAF12–MAGEA3 ternary complex to provide molecular insights into substrate recognition and binding. Several E3 ligases have recently been the focus of drug-discovery programs for targeted protein degradation. Our study provides the tools necessary to facilitate the discovery and development of DCAF12-based proteolysis targeting chimeras (PROTACs).

## Introduction

Protein degradation is a key housekeeping mechanism that allows for proper cell function and homeostasis maintenance. The ubiquitin-dependent proteolysis system is essential for protein turnover and relies on target protein ubiquitination as a prior step to proteasome-dependent degradation (1). The Cullin–RING ligases (CRLs) are part of the largest family of ubiquitin ligases in eukaryotes, with extensive variability of protein members and degradation targets (2, 3). The family shares a conserved architecture in which Cullin proteins—named from CUL1 to CUL7—serve as a scaffold for E2 and E3 ligases, as well as RING finger and adaptor proteins (2, 3). The Cullin E3 ligases are responsible for recruiting proteins for degradation, while the E2 ligases regulate ubiquitin transfer to the target protein.

Damaged DNA-binding protein-1 (DDB1)- and CUL4-associated factor 12 (DCAF12) is part of the CUL4 E3 ligase complex, which utilizes DDB1 as the adaptor protein linking Cullin4 to the substrate recognition module (4). While the biological function of DCAF12 remains relatively understudied, recent reports have associated it with neurotransmitter release (5) and spermatogenesis (6). DCAF12 recognizes the C-terminal double-glutamic acid (-EE) residues of substrate proteins, thereby participating in assembly quality control by recognizing incomplete or incorrectly assembled protein complexes for recruitment into the E3 ligase complex, leading to ubiquitination, and subsequent degradation via the ubiquitin proteasome complex (7, 8).

Various C-terminal “degrons,” transposable degradation causing sequences, have been reported to regulate the stability of many proteins via interactions with Cullin–RING E3 ligases, including KLHDC2, KLHDC3, and KLHDC10, that recognize a C-terminal di-glycine motif (9). More recently, the workhorse targeted protein degradation (TPD) E3 ligase CRBN has been reported to recognize C-terminal cyclic imides, posttranslational modifications that arise from intramolecular cyclization of glutamine or asparagine residues, as physiological degrons on substrates (10, 11).

DCAF12 protein is a member of the WD40-repeat (WDR) family and shares the characteristic β-propeller/donut-shaped fold (12). The WDR protein family has recently emerged as a promising target class for drug discovery, as several members of the family are implicated in key biological functions and have good druggability scores (12, 13). In addition, E3 ligases and several other proteasome-related proteins have been in the spotlight for their potential to expand the druggable proteome by enabling TPD (14, 15). The TPD strategy involves hijacking the E3 ligase function by creating small molecule degraders—molecular glues or proteolysis targeting chimera (PROTACs)—that bind to the E3 ligase and recruit proteins of interest for proteasome-dependent degradation (1, 16). DCAF12 is highly expressed, involved in K48 ubiquitination, and has a druggable WDR domain, which makes it an attractive E3 ligase for TPD.

In light of recent findings elucidating the importance of C-terminal -EE degron for DCAF12-substrate recognition and binding to TPD (7), we decided to further characterize the DCAF12-substrate interaction both in vitro and in cell. Previous studies have shown that the presence of DCAF12 is directly linked to the decrease in melanoma-associated antigen 3 (MAGEA3) and CCT5 (T-complex protein 1 subunit epsilon) protein levels in cells (7).

MAGEA3 is a member of a MAGE family of about 60 genes with conserved MAGE domain that are known to regulate RING-type E3 ligase activity by directly interacting with the RING ligase domain (17). Even though MAGEA3 is normally expressed in male germ cells, the protein is also aberrantly expressed in various cancer types, including breast and lung cancer (18), multiple myeloma (19), and melanoma (20, 21). Despite its implications for tumorigenesis (17) and poor patient prognosis, the biochemical or structural characterization of MAGEA3 interaction with DCAF12 has not been reported. On the other hand, CCT5 is a component of a Chaperonin-containing T-complex (TriC), which is a large (∼1 MDa) cylindrical-shaped multiprotein complex involved in the folding of nearly 10% of the human proteome (22).

To enable the discovery of small molecule modulators/handles targeting the understudied DCAF12 E3 ligase, we sought to develop a toolkit by structurally and biochemically characterizing the interaction between the WDR domain of DCAF12 and its target degrons. In this study, we show that DCAF12 can be successfully expressed and purified in vitro together with other members of the E3 ligase complex (DDB1 and DDA1) that are suitable for assay development and structural studies. We developed a suite of biophysical assays, to characterize the binding of both MAGEA3 and CCT5 C-terminal degron peptides to the DCAF12 WDR domain alone and in complex with DDB1 and DDA1. We also developed a cellular proximity-based assay to evaluate its target engagement in cells. Finally, we generated a cryo-EM structure of DDB1–DCAF12–MAGEA3 complex, revealing that the MAGEA3 C-terminal degron interacts with the conserved arginine and lysine residues located in the central cavity of DCAF12 WDR domain.

## Results

### DCAF12 binds to MAGEA3 and CCT5 C-terminal degron with high affinity in vitro

DCAF12 is a member of the human CRL4 E3 ubiquitin ligase complex that is known to interact with the adaptor protein called DDB1 and acts as the substrate recognition component of the E3 ligase complex. To characterize the DCAF12 interaction with the reported substrates, we expressed the DCAF12 protein alone, and in complex with DDB1 and DDA1. Despite our attempts to express and purify the full-length (FL) DCAF12 in isolation, we were only able to produce the N-terminally truncated form of DCAF12 (Δ1–78) in our hands (Fig. S1A). Soluble expression of DCAF12-FL was achieved only in the presence of DDB1, resulting in improved protein stability and purification yield (Fig. S1A and B).

Previous studies have shown DDA1 as a core component of CRL4 E3 ligase complex, interacting with DDB1 (23, 24) and making additional contacts with other members of DCAF substrate receptor proteins, as shown in DDB1–DCAF15–DDA1 structure (25–27). To investigate the potential role of DDA1 in DCAF12–DDB1 complex stability, we co-expressed and co-purified His-tagged DCAF12-FL together with untagged DDB1-FL and DDA1-FL. The intact mass spectrometry analyses confirmed the complexation of DDA1 together with DCAF12 and DDB1 after protein purification (Fig. S1A–C).

**Fig. 1.**
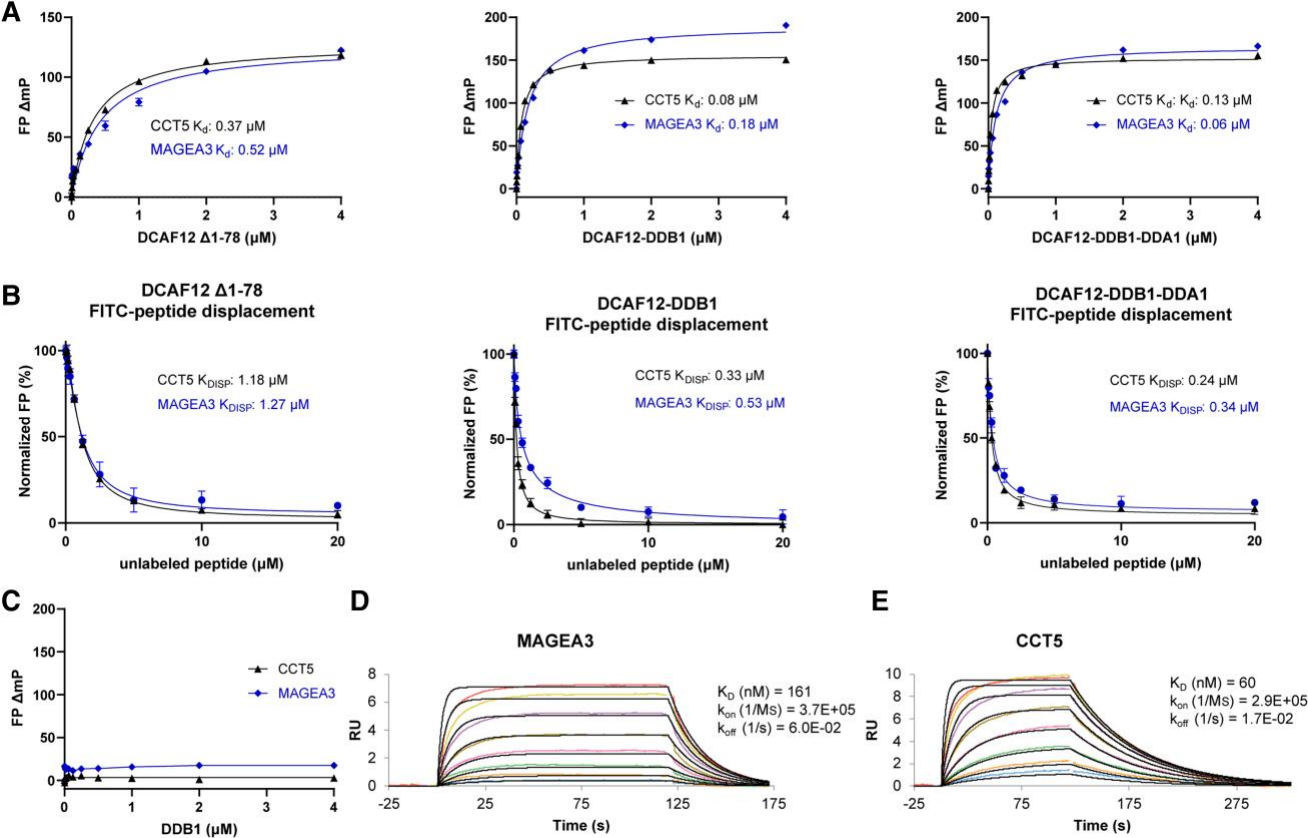
In vitro studies of DCAF12 interaction with MAGEA3 and CCT5 peptides. A) MAGEA3 and CCT5 peptides show similar binding affinities to DCAF12 alone and in complex with DDB1 and DDA1 in FP peptide binding assay. B) FP peptide displacement studies of DCAF12 bound to FITC-labeled MAGEA3 and CCT5 peptides with increasing concentrations of unlabeled peptides. C) CCT5 and MAGEA3 peptides show no binding to DDB1 when tested in FP assay. D and E) SPR analysis showing tight and similar binding of MAGEA3 and CCT5 peptides to biotinylated DCAF12 (Δ1–78).

To further characterize DCAF12 binding to MAGEA3 and CCT5 degrons, we developed a fluorescence polarization (FP)-based peptide binding and displacement assay in vitro. We designed small N-terminal fluorescein isothiocyanate (FITC)-labeled peptides harboring 11 C-terminal residues of MAGEA3 (LHEWVLREGEE) and nine from CCT5 (IRKPGESEE), both carrying the known -EE degron. In our FP direct binding assays, both MAGEA3 and CCT5 peptides showed potent binding to DCAF12 (Δ1–78), DDB1–DCAF12, and DDB1–DCAF12–DDA1 complex, with *K_d_* values in the nanomolar range for both peptides (Fig. 1A, Table S1).

Binding specificities were also evaluated by displacing the FITC-labeled peptides with the corresponding unlabeled peptides in a concentration dependent manner. Both FITC-labeled MAGEA3 and CCT5 peptides were successfully displaced in our assays by increasing concentrations of unlabeled peptides, confirming specific and reversible peptide–protein binding (Fig. 1B, Table S1).

To rule out any direct involvement of DDB1 within the DCAF12–DDB1 complex in interaction with MAGEA3 and CCT5 peptides, we tested the direct binding activity of DDB1 alone in our FP assay using the same peptides. No interaction was observed between DDB1 alone and any of the tested peptides (Fig. 1C), confirming that the peptides interact directly with DCAF12 only. In addition, we also tested the FP binding activity of DCAF12 on a control peptide derived from Histone H3 K9A mutant variant (H3K9A) (ARTKQTARASTGGK), which lacks the -EE residues at its C-terminal end. DCAF12 did not show any binding to the control peptide, thus ruling out any nonspecific interaction between the FITC label and DCAF12 (Fig. S2).

To further validate our FP assay results, we developed a surface plasmon resonance (SPR) assay using biotinylated DCAF12 (Δ1–78) immobilized on an SPR chip, with unlabeled MAGEA3 and CCT5 peptides titrated against it; both peptides showed similar binding affinities comparable to the FP assay (Fig. 1D and E).

### Conserved residues on the DCAF12 central channel are essential for binding to C-terminal degrons of MAGEA3 and CCT5 in cells

Previous studies have shown that MAGEA3 and CCT5 expression is directly influenced by the presence of DCAF12, with DCAF12 knockout cells presenting higher expression of both proteins (7). In addition, the presence of two glutamic acid residues at the protein C-terminal end is a key for MAGEA3 and CCT5 degradation, suggesting a direct role in protein–protein interaction with DCAF12 E3 ligase (7).

To further investigate the DCAF12 interaction with MAGEA3 and CCT5 in cells, we developed a NanoBRET assay, which measures the interaction of NanoLuc luciferase-tagged DCAF12 (energy donor) with fluorescently labeled HaloTag-tagged (energy acceptor) FL MEAGEA3/CCT5 or C-terminal peptide containing degron sequences (LHEWVLREGEE/IRKPGESEE, respectively). When donor and acceptor proteins are in close proximity, the donor excites the acceptor, which then emits fluorescence, allowing for direct measurement of protein–protein interaction in cells.

**Fig. 2.**
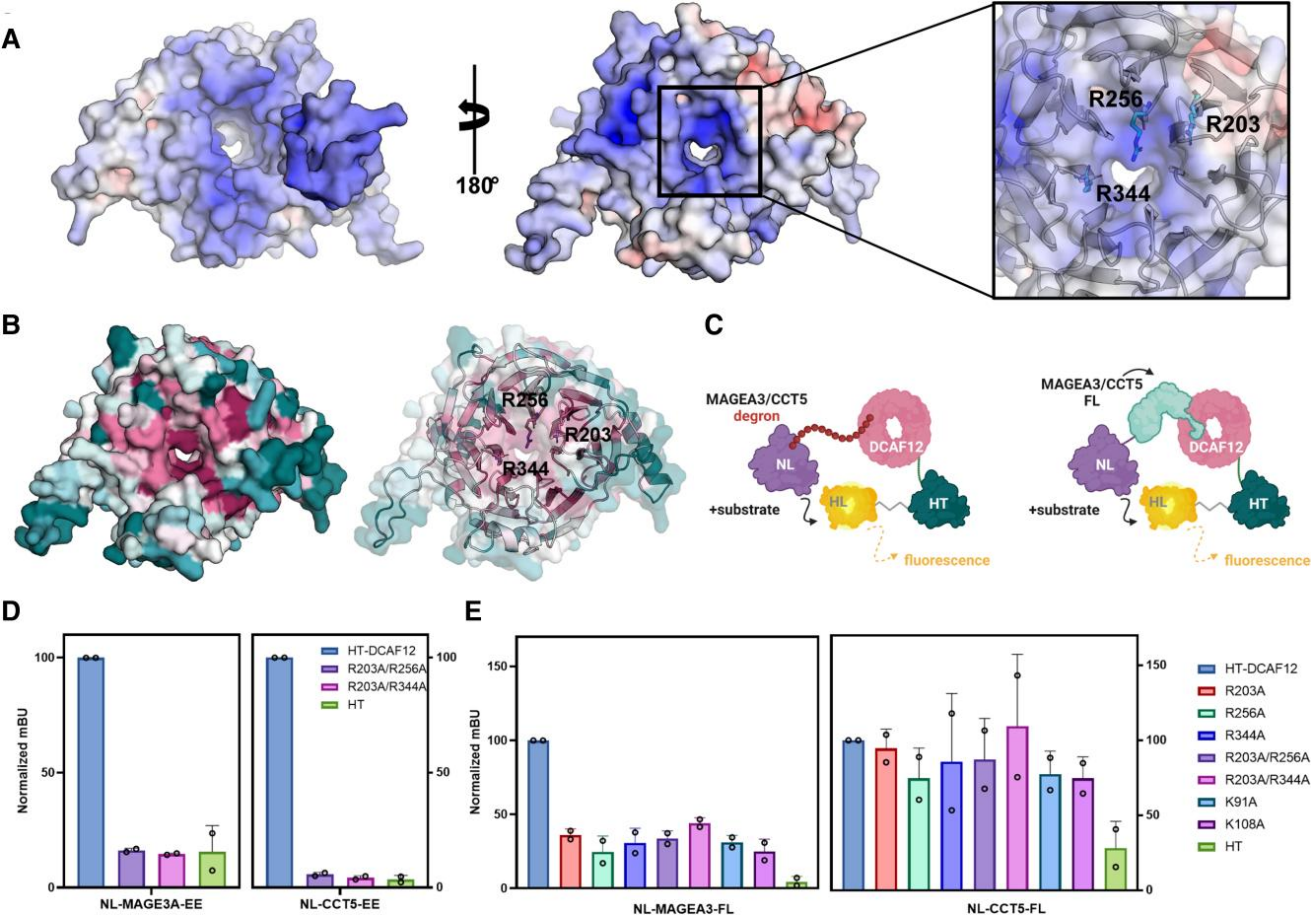
Conserved arginine and lysine residues near DCAF12 central channel participate in MAGEA3 binding in cells. A) DCAF12 electrostatic surface potential analysis showing negatively and positively charged residues colored in red and blue, respectively. On the left, DDB1 contacting surface of DCAF12. On the right, substrate contacting surface with positively charged residues—Arg203, Arg256, and Arg344—highlighted as sticks. Electrostatic potential is shown on a scale of −10 (red) kT/e to 10 (blue). B) Conservation analysis of DCAF12 showing high conservation of the same positively charged residues (represented as sticks) among homologous sequences. Residues were colored on a scale from variable/low conservation (dark teal) to high conservation (magenta). C) Schematic of NanoBRET strategy using MAGEA3/CCT5-FL protein or degron sequence cloned in frame with NanoLuc. DCAF12-WT and mutants were cloned in frame with HaloTag. D) NanoBRET assay using degron sequences of MAGEA3 and CCT5 against DCAF12-WT and combined arginine mutants R203A/R256A and R203A/R344A. E) Effect of DCAF12 mutations K91A, K108A, R203A, R256A, and R344A on the interaction with CCT5 and MAGEA3-FL proteins in NanoBRET assays in HEK293T cells.

To demonstrate the specific interaction between MAGEA3/CCT5 and DCAF12 WDR domain in cells, we explored suitable DCAF12 mutants in the WDR central pocket that can disrupt the interaction with -EE degrons. As no structure was available for DCAF12 at the time of our initial analysis, we took advantage of the predicted AlphaFold (28, 29) structure; cryo-EM structures of CCT5 bound to DCAF12 were recently published during writing of this manuscript (26). Considering the negative charge of the -EE side chains, our prediction was that the interaction between MAGEA3/CCT5 and DCAF12 would involve electrostatic interactions with positively charged, conserved residues within the central pocket of the DCAF12 WDR domain. A combination of electrostatic potential and sequence conservation analyses revealed that three arginine residues, Arg203, Arg256, and Arg344, have key contributions to the highly positive surface charge of DCAF12 (Fig. 2A and B).

Therefore, we used site-directed mutagenesis to generate two DCAF12 mutants in the central pocket of the WDR domain: R203A/R256A and R203A/R244A. DCAF12 wild-type (WT) and mutant interactions with MAGEA3 and CCT5 degron peptides were tested in a NanoBRET proximity-based assay (Fig. 2C). Both DCAF12 R203A/R256A and R203A/R244A mutations significantly decreased DCAF12 interaction with MAGEA3 and CCT5 degron peptides, confirming the interaction is specific and the peptides bind to the central pocket of DCAF12 WDR domain (Fig. 2D).

To evaluate the ability of DCAF12 to interact with FL MAGEA3 and CCT5 proteins in cells, we repeated the assay using the FL proteins instead of the degron peptides. In addition to the DCAF12 arginine mutants, the K91A and K108A mutants were also included in our assays, as both residues were shown to coordinate CCT5 binding to DCAF12 in a recently published DDB1–DCAF12–CCT5 cryo-EM structure (30). Interestingly, although all the mutations decreased DCAF12 interaction with MAGEA3-FL by over 50%, no changes were observed in its interaction with CCT5-FL (Fig. 2E). Overall, the NanoBRET assay results confirmed that positively charged residues located near the central channel of DCAF12 WDR domain are critical for the interaction with C-terminal -EE degron. In the future, the assay can be used to determine the target engagement of small molecules that disrupt the interaction between DCAF12 and its substrates in cells.

### DCAF12 interaction with MAGEA3 relies on C-terminal -EE degron

**Fig. 3.**
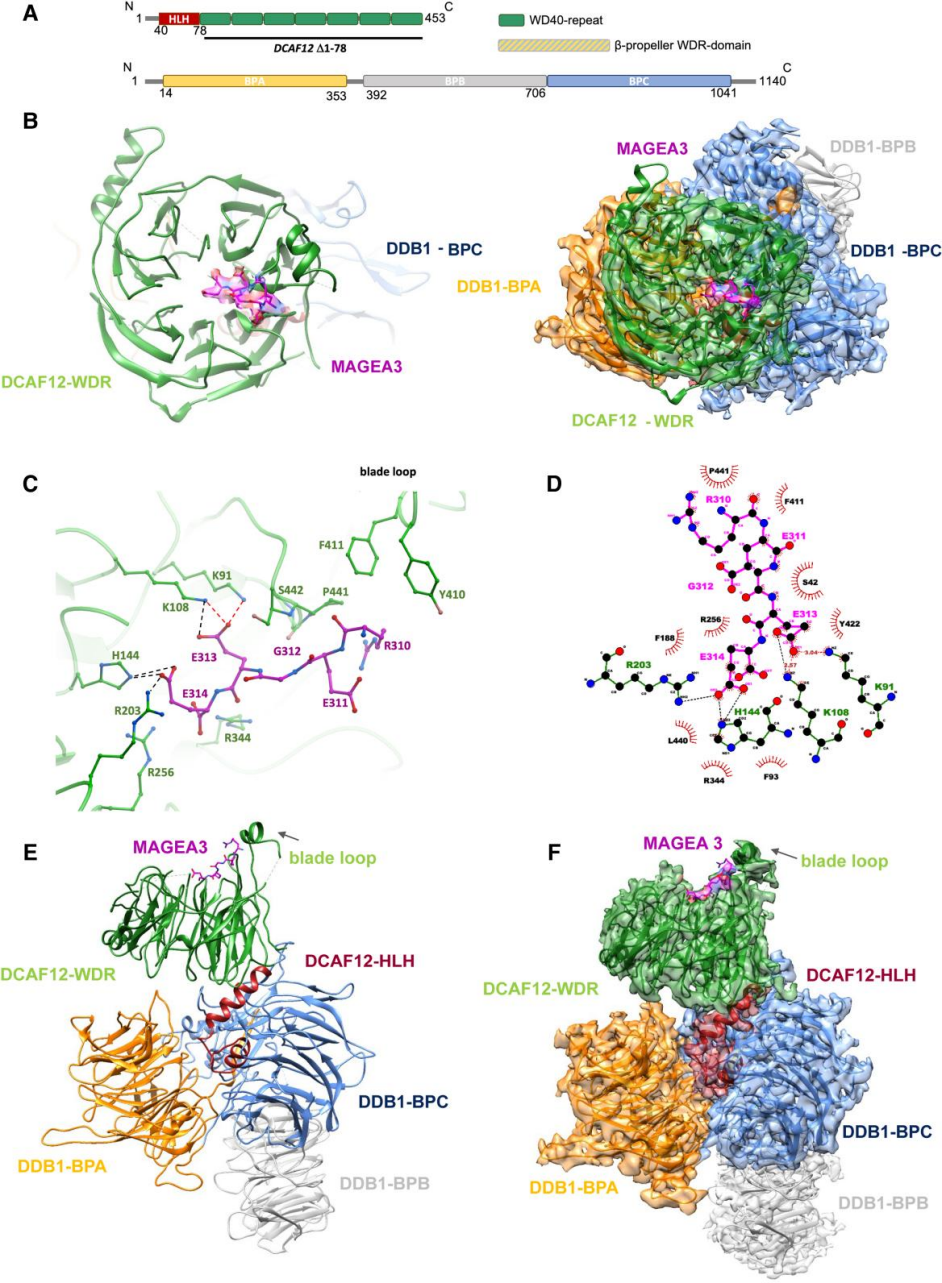
DDB1–DCAF12–MAGEA3 cryo-EM structure. A) Schematic of DCAF12 and DDB1 secondary structures with structural domains highlighted. B) Left panel, cryo-EM structure of DCAF12-FL bound to MAGEA3 peptide. DCAF12 is represented as cartoon and MAGEA3 as sticks. Right panel, frontal view of DDB1–DCAF12–MAGEA3 cryo-EM maps with model fitted. C) A close-up view of the interaction site between DCAF12 (green) and the MAGEA3 C-terminal degron peptide (magenta). Key DCAF12 residues that are involved in MAGEA3 peptide binding are represented as green sticks. Hydrogen bonds and salt bridges are shown in red and black dashed lines, respectively. D) Ligplot diagram of DCAF12 interactions with MAGEA3. MAGEA3 peptide residues are highlighted in magenta. DCAF12 residues making hydrogen bonds (red dashed lines) and salt bridges (black dashed lines) are colored green. DCAF12 residues involved in hydrophobic contacts are represented as red semicircles. Analysis was performed using LigPlot^+^ (31). E, F) Side view of DDB1–DCAF12–MAGEA3 cryo-EM model (left) and model fitted into maps (right).

To structurally characterize the DDB1–DCAF12 interaction with MAGEA3, we generated the 3.17 Å cryo-EM structure of the DDB1–DCAF12–MAGEA3 complex. The structure confirms the seven-bladed β-propeller fold of DCAF12 (residues 79–453), characteristic of the WDR domain (12) (Fig. 3A and B). The interaction with MAGEA3 is driven by the DCAF12 WDR domain, wherein the C-terminal degron residues of MAGEA3 interact with the DCAF12 central pocket (Fig. 3B and C). Interestingly, although the MAGEA3 protein was co-expressed and co-purified as a 212-amino acid construct, only the last five C-terminal residues (REGEE) were observed in our cryo-EM map (Figs. 3B–D and S3), suggesting that MAGEA3 interacts with DCAF12 solely through its C-terminal disordered degron end.

A closer look into the DCAF12–MAGEA3 interaction site reveals that the C-terminal Glu313 and Glu314 residues of MAGEA3 are anchored to the DCAF12 central pocket, while the N-terminal portion extends toward a small loop (residues 438–447) positioned above the central pocket (Fig. 3C). The central pocket of DCAF12 has two distinct patches of positively charged residues that play a crucial role in substrate recognition and binding. Notably, Arg256 and Arg344 residues enhance substrate binding by establishing interactions with the Glu314 backbone of MAGEA3 (Fig. 3C and D). The binding of MAGEA3 Glu314 to DCAF12 is further stabilized by salt bridges with Arg203 and His144 residues (Fig. 3D). On the other hand, Lys91 and Lys108 residues interact with Glu313 of MAGEA3 through a combination of hydrogen bonds and salt bridges (Fig. 3C and D).

The N-terminal portion of the MAGEA3 degron peptide interacts with DCAF12 through several hydrophobic residues. The three-carbon aliphatic straight chain of Arg310 of MAGEA3 participates in hydrophobic interactions with Tyr410, Phe411, and Pro441 residues of DCAF12 (Fig. 3C and D).

The cryo-EM structure also shows the conserved folding of DDB1, formed by three β-propeller domains (BPA, BPB, and BPC; Fig. 3E and F). DDB1-BPA and DDB1-BPC play a crucial role in anchoring DCAF12, which relies on a helix-loop-helix region in the DCAF12 N-terminus (Fig. 3A and E). The DDB1-BPB β-propeller domain interacts with the CUL4 scaffold protein in the E3 ligase complex and does not make any contact with DCAF12 or other DCAFs (Fig. 3E and F).

## Discussion

E3 ligases are essential for cell homeostasis maintenance and protein turnover, as they function as key components of the ubiquitin/proteasome-dependent protein degradation system. Despite increasing interest in this target class for TPD, the great majority of E3 ligases remain underexplored for PROTAC development (32).

Ligases with broad target repertoires might be more likely to recognize neo-substrates, making DCAF12 a promising E3 ligase for TPD due to its ability to interact with various substrates by recognizing and binding to short peptide sequences (C-terminal degrons) of target proteins, such as CCT5 (7, 30), MAGEA3 (7, 8), and the RNA helicase MOV10 (6). This interaction is mediated through the central pocket of DCAF12 WDR domain that could be targeted with small molecule handles for PROTAC design and development.

Previous studies have reported DCAF12-dependent degradation of GFP-peptide fusion bearing -EE-end degron, as well as MAGEA3 and CCT5 endogenous proteins in cells (7). Our biophysical data demonstrates that DCAF12 interacts with MAGEA3 and CCT5 degron peptides with nanomolar affinity in vitro. The similarity in binding affinities (<5-fold difference) for DCAF12 alone, and in complex with DDB1/DDA1 suggests that DCAF12 WDR domain is directly responsible for degron binding, with no contribution from DDB1. Moreover, the full displacement of labeled degron peptides upon titration of unlabeled peptide confirms binding specificity and suitability as a tool compound for small molecule screening. A recent study reported CCT5 peptide binding to DCAF12–DDB1 complex using a TR-FRET assay with *K_d_* and *K*_DISP_ values similar to our affinity determinations (<3-fold difference) (30). To our knowledge, there are no current reports of biophysical characterization of MAGEA3 and DCAF12 interaction other than the one provided by our study.

Our NanoBRET assay results suggest that the WDR central channel of DCAF12 is responsible for interaction with the degron peptides, mediated by positively charged residues. The central pocket of other WDR proteins, such as DCAF1, WDR5, and EED, has previously been reported as highly druggable and implicated in protein–protein interactions (12). The nature of the DCAFs interaction with DDB1 through the N-terminal region occludes one side of the DCAFs central channel, leaving exposed for interactions only the upper side, as demonstrated by our cryo-EM data and previous DCAFs/DDB2–DDB1 structures (25, 33, 34).

Intriguingly, although mutations on the DCAF12 central channel impaired the interaction with MAGEA3 degron peptide and FL protein in our NanoBRET assay, only CCT5 degron peptide responded to DCAF12 mutations. Our analysis shows that CCT5-FL has low engagement with DCAF12 in cells, with a 3.6-fold higher BRET signal than the negative control, compared with 22-fold difference in signal from MAGEA3-FL. Moreover, DCAF12 mutations have little to no effect on their interaction, even when residues known to coordinate their binding were mutated (30). CCT5 protein belongs to a chaperonin family, and it is known to form an oligomeric complex containing two rings with eight copies of the protein each (22). Interestingly, the CCT5 C-terminal degron residues from both rings seem to point toward the solvent-protected region of the oligomer (Fig. S4), therefore making the -EE residues possibly inaccessible for interactions. Endogenous CCT5 protein levels were previously shown to increase upon DCAF12 knockout in the A-375 cancer cell line (7), but the interaction could not be confirmed in our analysis using HEK293T cells. As shown recently, only monomeric CCT5, but not CCT5 assembled into a TRiC chaperonin complex could be ubiquitinylated by DCAF12 (30). Therefore, the CCT5 degron may be present in monomeric form and exposed for interaction with DCAF12 only in certain circumstances in cells. In line with this hypothesis, DCAF12-mediated MAGEA3 ubiquitination also takes place only under starvation conditions (8).

Finally, our cryo-EM structure reveals a stable complex formation between DCAF12 and MAGEA3, previously identified as a DCAF12 degradation target (7, 8). The structure highlights the critical role played by the C-terminal double-glutamic acid residues (Glu313 and Glu314) of MAGEA3 in its recognition and binding to DCAF12. Notably, the positively charged residues, such as Arg203, Arg256, Arg344, Lys91, and Lys108, located within the central channel of DCAF12, significantly contribute to the binding of MAGEA3 by directly interacting with the terminal Glu313 and Glu314 residues. The significance of these residues was further confirmed by our cellular NanoBRET assay, wherein mutations in DCAF12 resulted in a near-complete abolishment of interaction with both the MAGEA3 degron and the FL protein.

The terminal double-glutamic acid residues of CCT5 (Glu541 and Glu540) have also been shown to directly interact with the Arg256, Lys91, and Lys108 residues of DCAF12 (30). Further, both MAGEA3 and CCT5 degrons share structural similarities in their binding mode to DCAF12, suggesting the existence of a conserved binding mechanism between DCAF12 and its targets that is independent of sequences preceding the C-terminal di-Glu degron (Fig. S5).

Notably the structural basis of C-terminal degron recognition has also been elucidated in several other Cullin E3 ligase systems, such as the β-propeller-based KLHDC2, where high-resolution structures of the substrate-binding kelch domain have been determined in the presence of diGly-ending substrate peptides, revealing the atomic basis of recognition through hydrogen bonding with multiple backbone carbonyls of the degron peptide in a deep surface pocket (9).

Our cryo-EM structure also highlights the conserved binding mode between DCAF12 and DDB1, with the N-terminal region of DCAF12 making interactions with DDB1-BPC and the WDR domain of DCAF12 interacting with DDB1-BPA and DDB1-BPC. Similar binding mode was also observed for DCAF1 (33) and DCAF15 (26), as well as other DDB1 interactors (35).

Overall, our study provides valuable tools for the development of small molecules targeting the WDR domain of DCAF12 for PROTAC development (36). First, we report the recombinant production of DCAF12 alone and in complex with interaction partners. Second, our FP and SPR assays will enable the screening and validation of compounds targeting DCAF12 in mid-to-high throughput format. Last, our NanoBRET assay using CCT5/MAGEA3 degrons and MAGEA3-FL will support target engagement evaluation of DCAF12 binders in cells through protein proximity.

## Materials and methods

### Protein cloning, expression, and purification

DCAF12 constructs were subcloned from Mammalian Gene Collection (MGC) stock BC063823 into baculovirus expression vector pFBOH-MHL. For co-expression with DCAF12, DDB1 (MGC stock BC063823) and DDA1 (MGC stock BC000615) were subcloned into pFBOH-LIC vector lacking any protein tags. For testing DDB1-FL direct binding to peptides on FP assay, the protein was expressed using pFBOH-MHL vector with His-tag. All protein constructs were expressed in *Spodoptera frugiperda* (Sf9), as previously described (37).

For protein purification, cells were resuspended in base purification buffer, supplemented with protease inhibitor, 0.1% NP-40, and benzonase (see Table S2, for buffer details). Suspended cells were lysed by sonication and clarified by centrifugation. The supernatant was collected and incubated with nickel or cobalt resin for 1 h, under rotation at 4 °C. Beads and supernatant were placed in an open column for affinity purification. Beads were washed with 10 to 20 column volumes (CVs) of base buffer, followed by a 5 CVs wash using base buffer supplemented with 5 mM imidazole. Protein was eluted using 5 CVs of base buffer supplemented with 250 mM imidazole. Protein was further purified by size exclusion chromatography using base buffer. Purification samples were analyzed by sodium dodecyl sulfate–polyacrylamide gel electrophoresis. Clean fractions were pooled together, concentrated, and stored at −80 °C. Details about purification buffer and steps for each construct are summarized in Table S2.

### Fluorescence polarization assay

FP-binding assays were performed using 20 mM Tris pH 7.5, 150 mM NaCl, 5% glycerol, 0.5 mM TCEP, and 0.01% Triton buffer. All tested proteins were titrated from an initial 4 µM highest concentration, followed by a factor two serial dilution. N-terminally labeled FITC peptides (LHEWVLREGEE for MAGEA3, IRKPGESEE for CCT5, and ARTKQTARASTGGK for H3K9A) binding to proteins was tested at 20 nM final concentration. The assay was incubated for 45 min at room temperature, and plates were read using BioTek Synergy 4 (BioTek) plate reader. Prior to displacement assay, proteins at binding *K_d_* concentration were mixed with FITC peptides at 20 nM final concentration. Displacement was evaluated by using a titration curve of unlabeled peptide, with 40 µM as the highest concentration. Buffer, incubation time, and reading conditions followed FP-binding protocol. All FP analyses were performed in triplicates and, at least, two independent experiments. All *K_d_* and *K*_DISP_ values were calculated using GraphPad Prism version 9.0.

### SPR analysis

For SPR experiments, DCAF12 Δ1–78 sequence was subcloned into pFBD-BirA vector, expressed in *sf9* cells, and purified as described (Table S2). Biotinylated DCAF12 (Δ1–78) was immobilized to a level of ∼3,200 RU on a Biacore Series S streptavidin chip (Cytiva BR100531) using a Biacore 8K+ (Cytiva). Experiments were performed in a running buffer of 10 mM 4-(2-hydroxyethyl)-1-piperazineethanesulfonic acid (HEPES), pH 7.4, 150 mM NaCl (Cytiva BR-1006-70), 3 mM EDTA (Corning 46-034-CI), 0.005% P20 (Cytiva BR-1000-54), 5% Glycerol (Sigma G5516-1L), and 2% DMSO (Millipore Sigma MX1457-6). CCT5 and MAGEA3 peptides were injected over a period of 120 s at 30 μL/min followed by a dissociation phase of 200 s in an 8-point 2-fold dilution series with a top concentration of 1 μM. All steps were carried out at 15 °C. Reference and buffer-blank subtracted data were analyzed with the Biacore 8K Evaluation Software and fit to a 1:1 Langmuir model to obtain binding kinetics and *K_D_* values.

### DCAF12 electrostatic surface and conservation analyses

DCAF12 electrostatic surface potential was generated using APBS (38) webserver. DCAF12 AlphaFold (28) predicted structure was prepared using PARSE force-field configuration (39). Automatic multigrid calculation was used to generate DCAF12 electrostatic potential. Residue conservation analyses were performed using ConSurf (40) server and DCAF12 AlphaFold predicted structure. Multisequence alignment was done using MAFFT method, and homologs were selected from UNIREF90 using an identity cutoff of 35–95%. Conservation scores were calculated using the Bayesian method. PyMOL (Schrödinger, LLC) was used for further structure analysis and figure preparation.

### NanoBRET assays using DCAF12 mutants and -EE degrons

HEK293T cells were plated in 96-well plates (2 × 10^4^/well) and 4 h later transfected with 0.03 µg/well N-terminally HT-tagged DCAF12 (WT or mutants) or HT alone and 0.001 µg/well N-terminally NL-tagged CCT5 and MAGEA3 (FL or -EE degrons) using X-tremeGene HP transfection reagent (Roche), following the manufacturer's instructions. The next day, media were replaced with 40 µL of dulbecco's modified eagle medium: nutrient mixture F12 (DMEM/F12) [no phenol red, supplemented with 4% fetal bovine serum (FBS), penicillin 100 U/mL and streptomycin 100 µg/mL] ± HaloTag NanoBRET 618 Ligand (0.5 µL/mL, Promega). Four hours later, 10 µL of NanoBRET Nano-Glo Substrate solution (8 µL/mL Nano-Glo substrate (Promega) diluted in DMEM/F12 no phenol red, supplemented with 4% FBS, penicillin 100 U/mL, and streptomycin 100 µg/mL) was added, and the signal was read. Donor emission at 450 nm (filter: 450 nm/BP 80 nm) and acceptor emission at 618 nm (filter: 610 nm/LP) were measured within 10 min of substrate addition using CLARIOstar microplate reader (Mandel). Mean corrected NanoBRET ratios were determined by subtracting the mean of 618/460 signal from cells without NanoBRET 618 Ligand × 1,000 from the mean of 618/460 signal from cells with NanoBRET 618 Ligand × 1,000. All DCAF12 mutants and -EE degron constructs were produced using Q5 site-directed mutagenesis kit (New England Biolabs).

### Sample preparation for electron microscopy

The QuantiFoil Au 1.2/1.3 300 mesh grids were glow discharged using PELCO easiGlow Discharge Cleaning System. A total of 3 μL of protein sample was applied onto the EM grids at a final concentration of 0.2 mg/mL. Sample vitrification was carried out using Vitrobot (Thermo Fisher Mark IV) with the following settings: blot time 3 s, blot force 3, wait time 0 s, inner chamber temperature 4 °C, and a 100% relative humidity. The EM grids were flash-frozen in liquid ethane cooled by liquid nitrogen. Cryo-EM data were automatically collected on a 200-kV Thermo Scientific Glacios microscope controlled by EPU software. Micrographs were captured at a scope magnification of 105,000× by a Facon4 detector (Gatan) operated in the counting mode. During a 6-s exposure time, a total of 40 frames were recorded with a total dose of 40 e^−^/Å^2^. The calibrated physical pixel size was 0.948 Å for all digital micrographs.

### Cryo-EM image processing and three-dimensional structure reconstruction

Cryo-EM data collection and image quality were monitored by the cryoSPARC Live v3.2 (41) installed in a local workstation. The image preprocessing steps included patch motion correction, patch contrast transfer function (CTF) estimation, blob particle picking (50–150 Å diameter), and extraction, all performed simultaneously. A total number of 6,777 raw micrographs were recorded during a 2-day data collection session using Glacios microscope. Acceptable 2D classes were further used as templates for particle repicking. Two rounds of 2D image classification were performed, resulting in ∼1.2 million good particle images (Fig. S6A). Particles were used for 3D reconstruction. Four starting 3D models were calculated, with one major 3D class obtained. This major class was subjected to nonuniform refinement without any mask. A mask was then generated based on the output. Further local refinement ensued, utilizing the nonuniform refinement particles, their corresponding map, and the newly generated mask. The global CTF refinement step was executed against a specified 3D reference structure. Subsequently, another round of local refinement was implemented, using the same settings, with CTF refinement particles replacing the original particles, resulting in the generation of a 3.17-Å resolution map.

Detailed statistics about the cryo-EM experiments are summarized in Table S3 and Fig. S6C–E.

### Cryo-EM model building, refinement, and validation

Human DCAF12–DDB1–CCT5 cryo-EM structure (PDB 8AJN) was used as the initial model for atomic model building of the EM map. To create a starting model for the DCAF12–DDB1–MAGEA3 complex, each subunit of the previously solved protein complex was individually used for map fitting using UCSF Chimera (42). MAGEA3 degron peptide was modeled using CCT5-binding site as reference. Models were refined using PHENIX (43) and COOT (44).

## Supplementary Material

pgae153_Supplementary_Data

## Data Availability

The source data underlying Figs. 1A–E and 2D, E are deposited in Zenodo with DOI: 10.5281/zenodo.10737414. Cryo-EM density map for DDB1–DCAF12–MAGEA3 complex has been deposited in the Electron Microscopy Data Bank (EMDB) under the accession code EMD-41105. The atomic model has been deposited in the Protein Data Bank under the accession code 8T9A.

## Preprints

This manuscript was posted as a preprint on bioRxiv: doi: https://doi.org/10.1101/2023.08.15.553295.
