# Supplementary material for: Probing the CRL4DCAF12 interactions with MAGEA3 and CCT5 di-Glu C-terminal degrons
Source: PNAS Nexus. 2024 Apr 10;3(4):pgae153. doi: 10.1093/pnasnexus/pgae153 (PMC11044963; doi:10.1093/pnasnexus/pgae153)
Supplement: pgae153_Supplementary_Data [file pgae153_supplementary_data.docx]

**SUPPLEMENTARY MATERIAL**

**Probing CRL4^DCAF12^ interactions with MAGEA3 and CCT5 di-Glu C-terminal degrons**

**Authors list:**

Germanna Lima Righetto^1,2^, Yanting Yin^3^, David M. Duda^3^, Victoria Vu^1^, Magdalena M Szewczyk^1^, Hong Zeng^1^, Yanjun Li^1^, Peter Loppnau^1^, Tony Mei^1^, Yen-Yen Li^1^, Alma Seitova^1^, Aaron N. Patrick^5^, Jean-Francois Brazeau^4^, Charu Chaudhry^5^, Dalia Barsyte-Lovejoy^1,2^, Vijayaratnam Santhakumar^1*^, Levon Halabelian^1,2*^

**Affiliations:**

^1^ Structural Genomics Consortium, University of Toronto, Toronto, Ontario M5G 1L7, Canada

^2^ Department of Pharmacology and Toxicology, University of Toronto, Toronto, Ontario M5S 1A8, Canada

^3^ Structural & Protein Sciences, Therapeutics Discovery, Janssen Research and Development, Spring House, Pennsylvania 19044, United States

^4^ Discovery Chemistry, Therapeutics Discovery, Janssen Research & Development, LLC, 3210 Merryfield Row, La Jolla, California 92121, United States

^5^ Discovery Technology and Molecular Pharmacology, Therapeutics Discovery, Janssen Research & Development, LLC, Welsh & McKean Roads, Spring House, Pennsylvania 19477, United States

**Corresponding authors:**

* **Vijayaratnam Santhakumar**

Address: Structural Genomics Consortium, 101 College Street, Toronto, Ontario M5G 1L7, Canada

**Email:** [santha.santhakumar@utoronto.ca](mailto:santha.santhakumar@utoronto.ca)

Tel: +1 416-946-7842

* **Levon Halabelian**

Address: Structural Genomics Consortium, 101 College Street, Toronto, Ontario M5G 1L7, Canada

**Email:** [l.halabelian@utoronto.ca](mailto:l.halabelian@utoronto.ca)

Tel: +1 416-946-3876

**
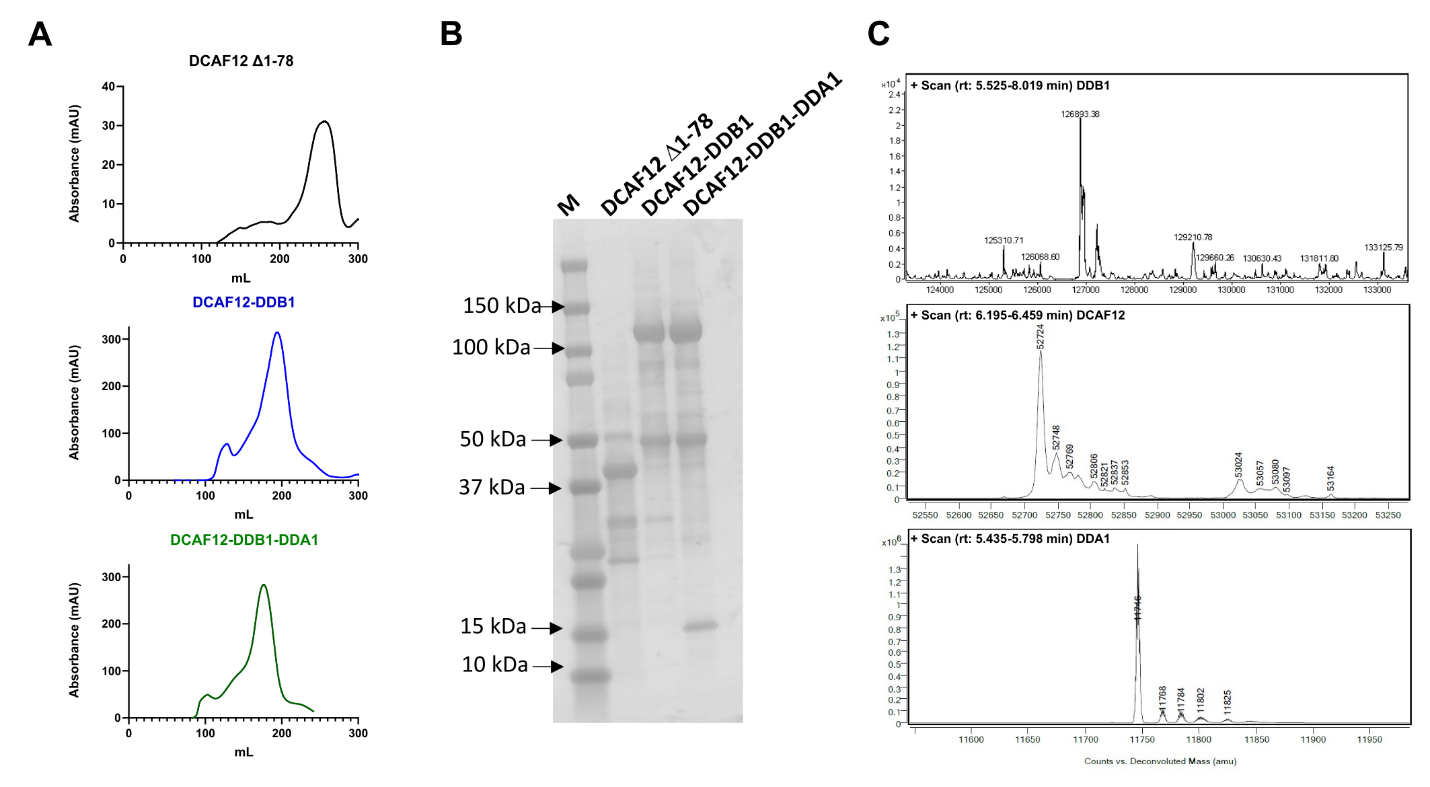
**

**Figure S1: Generation of DCAF12 in complex with DDB1 and DDA1. A:** Size exclusion purification profile of DCAF12 Δ1-78 (black), DCAF12-DDB1 (blue), DCAF12-DDB1-DDA1 (gray). **B:** SDS-PAGE analysis of final product of the size exclusion purifications shown in A. **C:** Intact mass LC-MS analysis of DCAF12-DDB1-DDA1 purification product showing peaks at expected molecular weight for each protein.


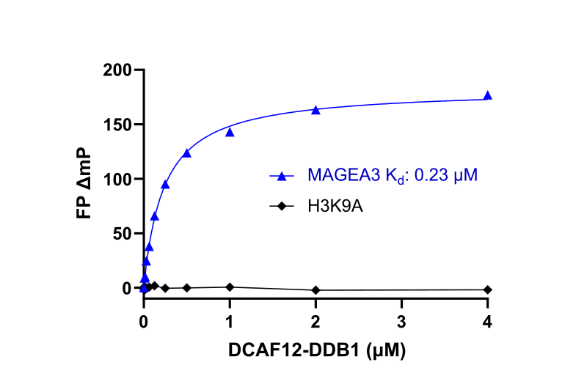


**Figure S2: DCAF12-DDB1 binding to unrelated peptide lacking -EE degron.** FP binding analysis comparing MAGEA3 and H3K9A binding to DCAF12-DDB1 complex.


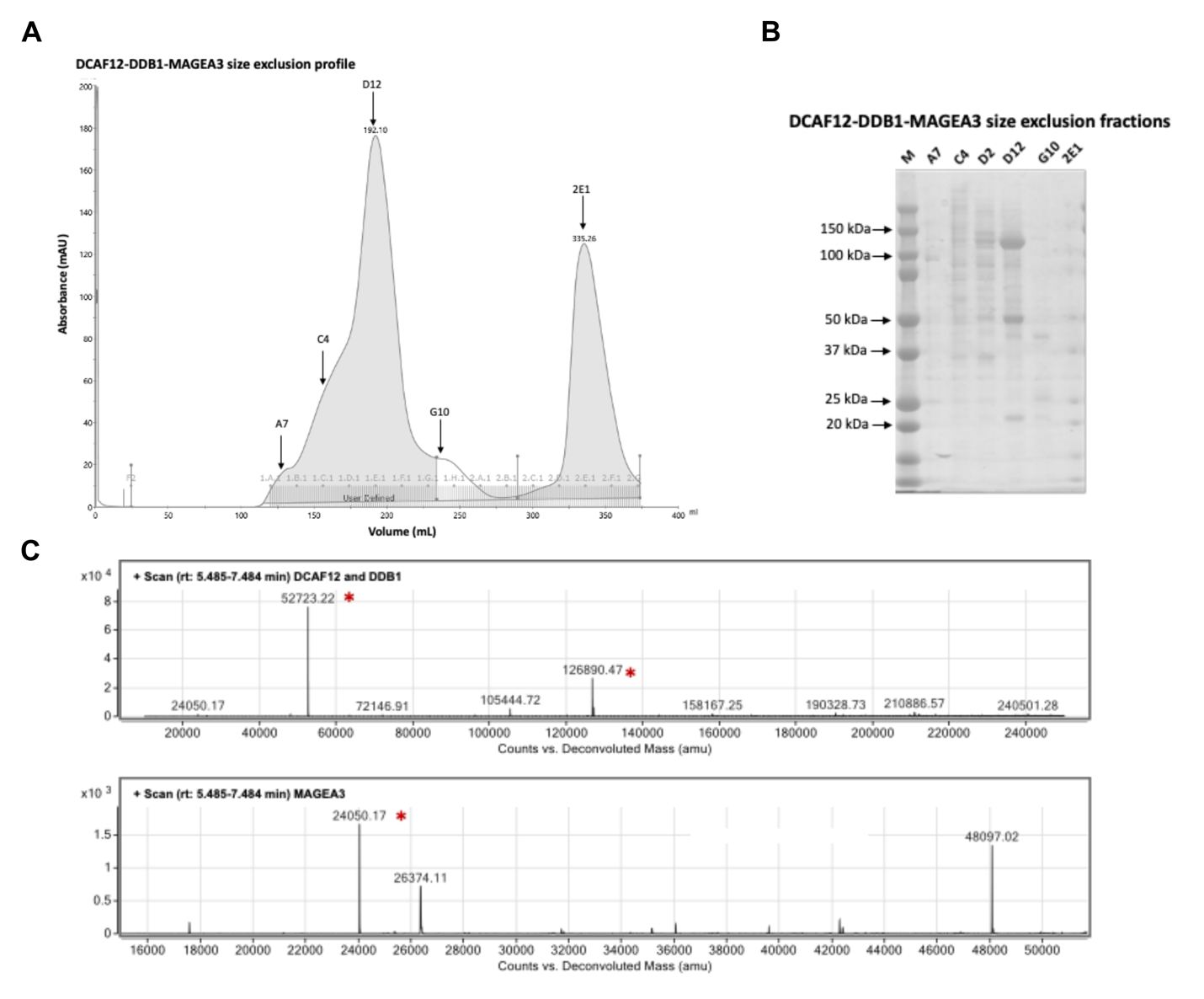


**Figure S3: Purification of DCAF12 in complex with DDB1 and MAGEA3. A:** Size exclusion purification profile of DCAF12 and DDB1 (FL) in complex with MAGEA3 (104-314). **B:** SDS-PAGE analysis of size exclusion samples. **C:** Intact mass LC-MS analysis of the final trimeric -complex after purification highlighting peaks at expected molecular weight for DCAF12, DDB1, and MAGEA3 proteins. Peaks of interest are highlighted with asterisks.

**
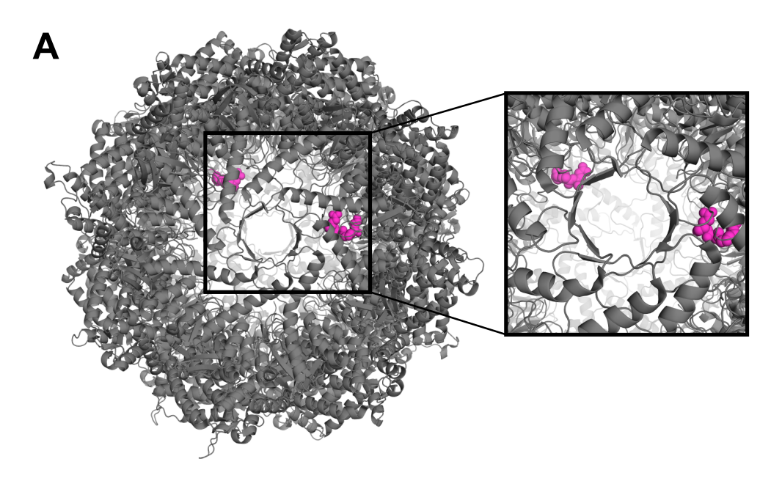
**

**Figure S4: CCT5 C-terminal -EE degron is buried in oligomeric TRiC/CCT structure.** Oligomeric CCT5 structure highlighting the position of C-terminal residues in the internal oligomer cavity (PDB ID: 7LUM). Terminal -EE residues are colored pink and shown as spheres.

**
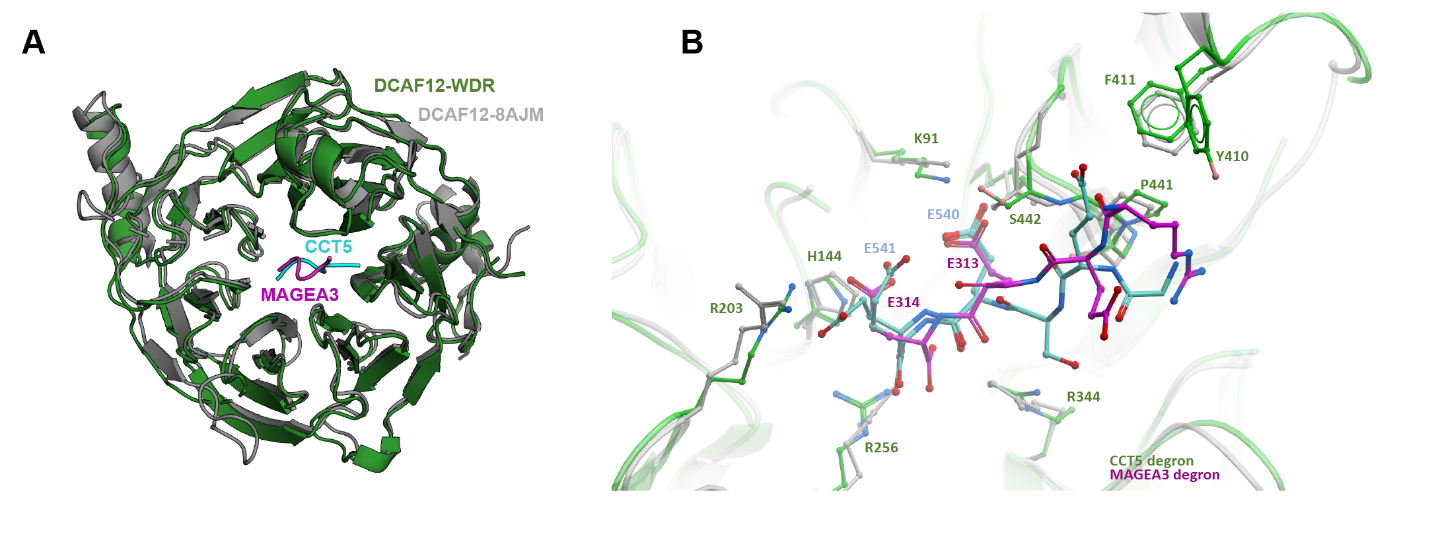
**

**Figure S5: DCAF12 binding to MAGEA3 and CCT5 comparison. A:** Structural alignment of DCAF12-MAGEA3 (green) and DCAF12-CCT5 structures (grey). Both MAGEA3 and CCT5 peptides bind DCAF12 in similar region, occupying part of the central channel. Alignment RMSD: 0.665 Å. **B:** Close-up of DCAF12 residues contacting MAGEA3 and CCT5 degrons. MAGEA3 peptide is shown as cartoon in magenta, CCT5 is shown in cyan. DCAF12-DDB1-CCT5 structure PDB ID: 8AJM.

**
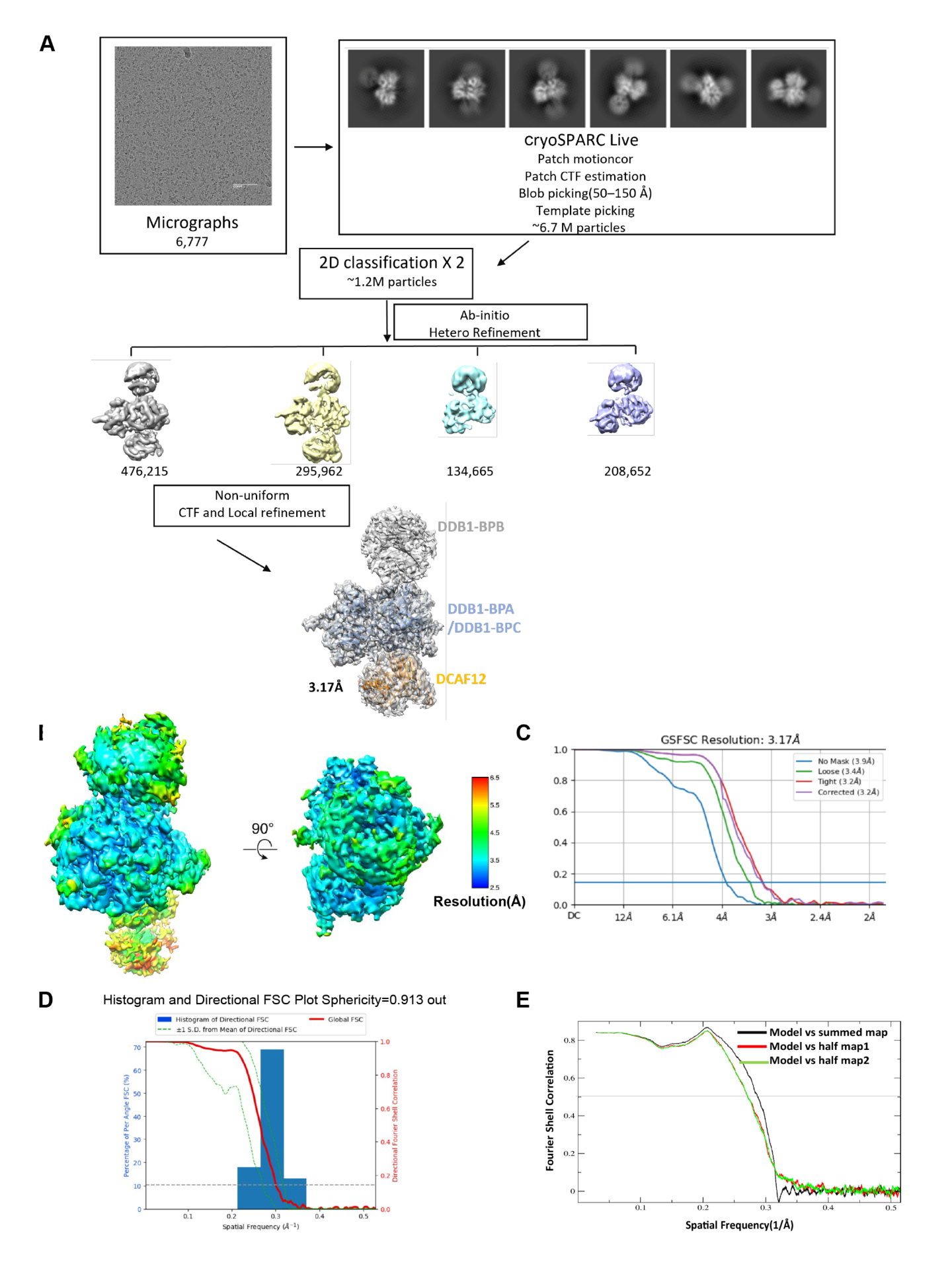
**

**Figure S6: DCAF12-DDB1-MAGEA3 Cryo-EM data processing and refinement strategy. A:** Workflow of image processing and 3D reconstruction. Representative micrograph from the DCAF12-DDB1-MAGEA3 collection is shown. Non-uniform analysis was applied to yield the 3.17-Å resolution 3D map. **B:** Resolution estimations of the DCAF12-DDB1-MAGEA3 complex. Local map resolution was estimated using ResMap program and colored as indicated. **C:** The 0.143 criterion of the gold standard Fourier shell correlation (GSFSC) was used to estimate the average resolutions. **D:** Map directional anisotropy quantification using 3D-FSC server (https://3dfsc.salk.edu/). The sphericity of complex is 0.913, demonstrating good anisotropic property of the map. **E:** Model validation by FSC curves comparison. In red, comparison between model and half map 1 (work). Model and half map 2 (free) comparison is colored in green, and model and full map is plotted in black.

**Table S1: Biophysical data summary**

| FP peptide binding | | | |
| --- | --- | --- | --- |
| Protein construct | Peptide | Kd (µM) | SD (µM) |
| DCAF12:79-453aa | CCT5 | 0.37 | 0.05 |
| DCAF12:79-453aa | MAGEA3 | 0.52 | 0.09 |
| DCAF12-DDB1 | CCT5 | 0.08 | 0.00 |
| DCAF12-DDB1 | MAGEA3 | 0.18 | 0.01 |
| DCAF12-DDB1-DDA1 | CCT5 | 0.13 | 0.02 |
| DCAF12-DDB1-DDA1 | MAGEA3 | 0.06 | 0.01 |
| FP peptide displacement | | | |
| Protein construct | Peptide | Kdisp (µM) | SD (µM) |
| DCAF12:79-453aa | CCT5 | 1.18 | 0.08 |
| DCAF12:79-453aa | MAGEA3 | 1.27 | 0.10 |
| DCAF12-DDB1 | CCT5 | 0.33 | 0.00 |
| DCAF12-DDB1 | MAGEA3 | 0.53 | 0.00 |
| DCAF12-DDB1-DDA1 | CCT5 | 0.24 | 0.03 |
| DCAF12-DDB1-DDA1 | MAGEA3 | 0.34 | 0.01 |
| SPR peptide binding | | | |
| Protein construct | Peptide | K_D_ (µM) | SD (µM) |
| DCAF12:79-453aa | CCT5 | 0.06 | 0.004 |
| DCAF12:79-453aa | MAGEA3 | 0.161 | 0.006 |

**Table S2: Protein purification information**

| Construct name | N-terminal tag | C-terminal tag | Purification step 1 | Purification step 2 | Final buffer | Application |
| --- | --- | --- | --- | --- | --- | --- |
| DCAF12 (Δ1-78) | MHHHHHHSSGRENLYFQG | N/A | Affinity purification, TALON beads | Superdex S200 26/60 | 20 mM Tris pH 8.5, 150 mM NaCl, 5% glycerol, 0.5 mM TCEP | FP assays |
| DCAF12 (Δ1-78) | MSGLNDIFEAQKIEWHEGSAGGSG | GGSGHHHHHH | Affinity purification, TALON beads | Superdex S200 26/60 | 20 mM Hepes pH 7.7, 250 mM NaCl, 5% glycerol, 0.5 mM TCEP | FP assays |
| DCAF12-FL; DDB1-FL | DCAF12: MHHHHHHSSGRENLYFQG | N/A | Affinity purification, Nickel beads | Superdex S200 26/60 | 20 mM Tris pH 7.5, 150 mM NaCl, 5% glycerol, 0.5 mM TCEP | FP assays |
| DCAF12-FL; DDB1-FL; DDA1-FL | DCAF12: MHHHHHHSSGRENLYFQG | N/A | Affinity purification, Nickel beads | Superdex S200 26/60 | 20 mM Hepes pH 7.4, 150 mM NaCl, 5% glycerol, 0.5 mM TCEP | FP assays |
| DCAF12-FL; DDB1-FL; MAGEA3 (Δ1-103) | DCAF12: MHHHHHHSSGRENLYFQG | N/A | Affinity purification, Nickel beads | Superdex S200 26/60 | 20 mM Tris pH 8, 150 mM NaCl, 0.5 mM TCEP | Cryo-EM |
| DDB1-FL | MGSSHHHHHHSSGLVPRGS | N/A | Affinity purification, TALON beads | Superdex S200 26/60 | 20mM Tris pH7.5, 250mM NaCl, 2mM TCEP, 2.5% propanediol | FP assays |

**Table S3: Data collection, reconstruction, model refinement, model-to-data fit statistics of Cryo-EM structures**

| **DCAF12-DDB1-MAGEA3**  **EMD-41105, PDB: 8T9A** | |
| --- | --- |
| **Data collection and processing** |  |
| Microscope | Glacios |
| Voltage (keV) | 200 |
| Nominal magnification | 105000 x |
| Exposure navigation | Image Shift |
| Electron exposure (e /Å^2^) | 40 |
| Total exposure time (sec) | 6 |
| Detector | Facon4 |
| Pixel size (Å)* | 0.948 |
| Defocus range (µm) | -0.6 to -2.0 |
| Micrographs Used | 6777 |
| Final Refined particles (no.) | 476215 |
| **Reconstruction** |  |
| Symmetry imposed | C1 |
| **Resolution** |  |
| FSC threshold 0.143 | 3.17 Å |
| **Refinement** |  |
| Protein residues | 1498 |
| R.m.s deviations |  |
| Bond lengths (Å) | 0.011 |
| Bond angles (°) | 0.975 |
| Ramachandran |  |
| Outliers | 0.00 |
| Allowed | 13.75% |
| Favored | 86.25 % |
| Rotamer outliers | 0.61 % |
| MolProbity score | 2.45 |
| Clash score | 20 |
| **Model-to-data fit** |  |
| CC(mask) | 0.78 |
| CC(box) | 0.78 |
| CC(peaks) | 0.72 |
| CC(volume) | 0.78 |
